# 744. Changing Molecular Epidemiology of Staphylococcus aureus Bacteremia in a Metropolitan Area in Canada

**DOI:** 10.1093/ofid/ofad500.805

**Published:** 2023-11-27

**Authors:** Thi Mui Pham, Joshua T Smith, Tatum D Mortimer, Dan Gregson, Soren Wacker, Bruce J Walker, Ashlee Earl, Ian Lewis, Yonatan H Grad

**Affiliations:** Harvard T.H. Chan School of Public Health, Boston, MA; Broad Institute of MIT and Harvard, Cambridge, Massachusetts; Harvard T.H. Chan School of Public Health, Boston, MA; University of Calgary, calgary, Alberta, Canada; University of Calgary, calgary, Alberta, Canada; Broad Institute of MIT and Harvard, Cambridge, Massachusetts; Broad Institute of MIT and Harvard, Cambridge, Massachusetts; University of Calgary, calgary, Alberta, Canada; Harvard Chan School of Public Health, Boston, Massachusetts

## Abstract

**Background:**

The incidence of *Staphylococcus aureus* bacteremia (SAB) has increased in Alberta over the past two decades. It is unclear whether this trend reflects the expansion of a single strain or multiple strains and how much it has been influenced by changes in antibiotic use and resistance.

**Methods:**

We used data from 5,881 *S. aureus* isolates representative of all SAB episodes from patients in the greater Calgary region from 2006-2019. Bacteremia episodes were defined as all positive isolates collected from a patient within a 30-day period. Each episode was categorized as community-onset (within 48 hours after admission) or hospital-onset (after 48 hours post admission). Isolates for which sequencing was successful (97.8%) were assigned strain and clonal complex (CC) designations. We computed the incidence of strains and antimicrobial susceptibility to cloxacillin, clindamycin, erythromycin, and ciprofloxacin. Antibiotic prescribing rates for the region were obtained from Alberta Health Services.

**Results:**

SAB incidence increased from 30 to 40 per 100,000 population. The prevalence of resistance to cloxacillin, clindamycin, erythromycin, and ciprofloxacin fluctuated and decreased from a maximum of 31.8% to 15.6%, 28.2% to 19.2%, 39.1% to 27.5%, and 37.4% to 15%, respectively, by the end of the study period. An overall increase in *S. aureus* antibiotic susceptibility was driven primarily by an increased incidence of susceptible sub-lineages within several strains (CC30, CC5, CC8, CC45, CC15, CC97) mainly causing community-onset SAB. The incidence of SAB resistant to all four antibiotics decreased over the study period due to a decline in community and hospital-onset infections by one resistant sub-lineage of CC5; from 4.1 to 0.3 per 100,000 population, and was associated with a decline in community antibiotic prescribing for beta-lactamase resistant penicillins, lincosamides, macrolides, and fluoroquinolones (from 6.07 to 3.07, 17.5 to 16, 77 to 73.6, and 55.6 to 40.3 age-standardized number of dispensation per 1,000 population).

Incidence rate of Staphylococcus aureus bacteremia per 100,000 Calgary residents stratified by community- and hospital-acquired infections.

**Figure:**
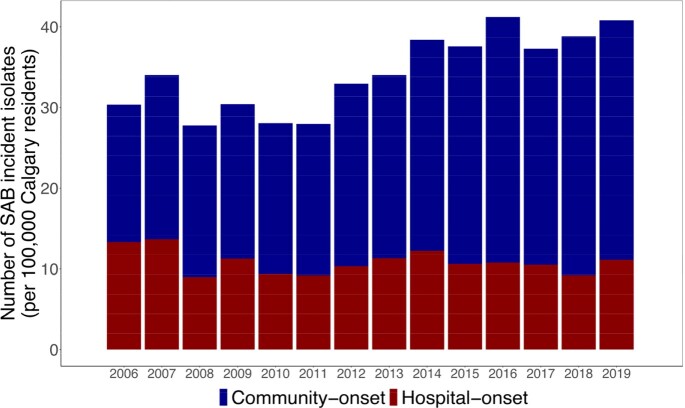

Bacteremia episodes were defined as all isolates collected from a patient within a 30-day period, and the incident isolate represents the first isolate from each episode.

Prevalence of resistance to cloxacillin, clindamycin, erythromycin, and ciprofloxacin between 2006 and 2019.

**Figure:**
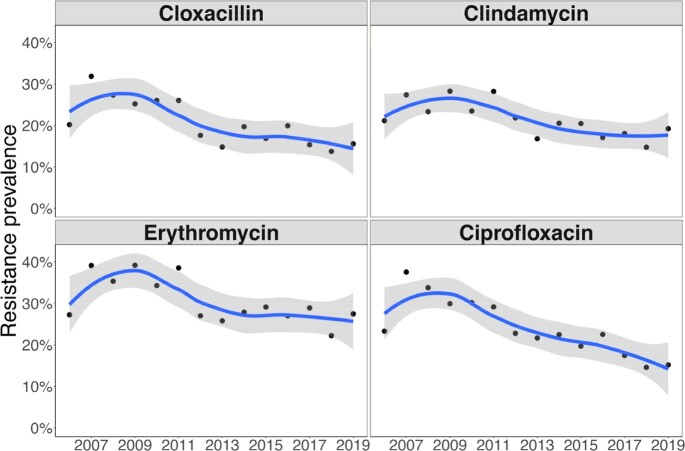

Points represent the proportion of incident isolates resistant to the respective antibiotic. Solid lines are smoothing curves using the loess method. Grey bands represent 95% confidence intervals.

Incidence rate of Staphylococcus aureus bacteremia per 100,000 Calgary residents stratified by onset of infection.

**Figure:**
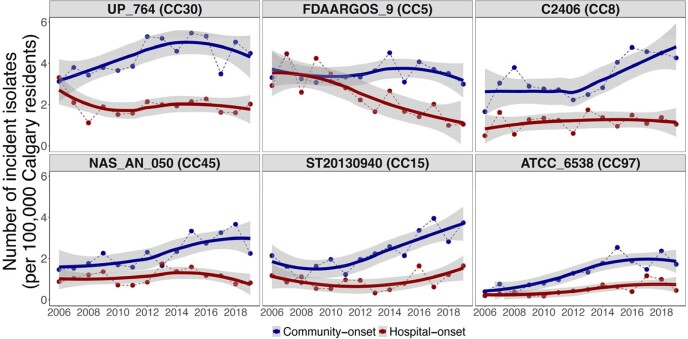

Points represent the incidence rate, dashed lines are linear interpolations between points, solid lines represent smoothing curves using the loess method. Grey bands represent 95% confidence intervals. Clonal complex designations in brackets represent the clonal complex assigned to the majority of the isolates for the respective strain.

**Conclusion:**

From 2006-2019, Calgary observed a decline in community antibiotic use, a reduction in the incidence of a resistant strain, and a greater increase in the incidence of community-onset SAB caused by several antibiotic susceptible strains.

**Disclosures:**

**Dan Gregson, MD**, BioMerieux Canada: Advisor/Consultant **Yonatan H. Grad, MD, PhD**, Day Zero Diagnostics: Board Member|GSK: Advisor/Consultant

